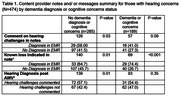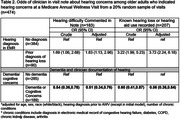# Discussion and Documentation of Hearing Difficulty by Providers in the Presence of Cognitive Concerns‐ study of the Medicare Annual Wellness Visit and Medial Record

**DOI:** 10.1002/alz.086742

**Published:** 2025-01-03

**Authors:** Danielle Powell, Stephanie Nothelle, Jamie Smith, Esther S Oh, Nicholas S Reed, Jennifer Wolff

**Affiliations:** ^1^ University of Maryland, College Park, MD USA; ^2^ Johns Hopkins School of Medicine, Baltimore, MD USA; ^3^ Johns Hopkins University, Baltimore, MD USA; ^4^ Johns Hopkins University School of Medicine, Baltimore, MD USA; ^5^ Johns Hopkins Bloomberg School of Public Health, Baltimore, MD USA; ^6^ Johns Hopkins University Bloomberg School of Public Health, Baltimore, MD USA

## Abstract

**Background:**

How clinicians discuss, document, and diagnose health concerns within a visit shapes patient perceptions of their health conditions. Undiagnosed hearing loss among older adults with dementia or cognitive concerns may exacerbate neuropsychiatric symptoms and care challenges. This study investigates clinician documentation of hearing concerns and whether documentation, diagnosis, and referral vary for older adults with dementia/cognitive concerns.

**Method:**

We use patient‐level health and demographic information from the electronic medical record and associated clinician visit notes for Medicare beneficiaries who indicated hearing concerns for the first time at an Annual Wellness Visit (AWV) between 2017‐2022 at a large academic health system. We analyzed the text of a random sample of AWV clinical notes to evaluate clinical documentation of hearing concerns/loss and placement of hearing‐related referrals. Logistic regression models investigate odds of clinician documentation of hearing concerns or diagnosis by diagnosed dementia (from ICD codes) or cognitive concerns (from AWV health risk assessment).

**Result:**

Among 2,593 beneficiaries who reported hearing concerns at an AWV (mean age 78.8 years, 56.6% female), 6.5% had a concurrent dementia diagnosis, nearly 40% indicated congruent cognitive concerns, and 20% had a prior diagnosis of hearing loss in the medical record. Text analysis (Table 1) of clinician visit notes from a random sample of patients (n = 474) found hearing concerns were less frequently documented for those with (versus without) diagnosed dementia (44.2% vs 30.2%). Clinicians were half as likely to comment on hearing (OR:0.51; 95%CI:0.34,0.76) or indicate known hearing loss in the AWV note (OR:0.56; 95% CI:0.38,0.84) for those with vs. without dementia (Table 2). Regardless of dementia status, about 8‐10% were recommended/received a referral to hearing care; only 50% with index hearing concerns received a diagnosis of hearing loss in the medical record following the AWV.

**Conclusion:**

Clinicians are less likely to document hearing concerns and incorporate hearing concerns as a health problem at the AWV for patients with dementia. The complex care needs of dementia may inhibit clinician ability to attend to other patient health concerns‐ like hearing. Opportunity to embed hearing and communication educational interventions which support clinicians in addressing patient‐collected health risks can improve overall dementia care.